# An unusual complication after transcatheter aortic valve implantation: a case report

**DOI:** 10.1093/ehjcr/ytae045

**Published:** 2024-02-02

**Authors:** Alba Abril Molina, Mónica Fernández Quero, José E López Haldón, Manuel Villa Gil Ortega, José F Díaz Fernández

**Affiliations:** Cardiology Department, Juan Ramón Jiménez University Hospital, Ronda Norte, s/n, Huelva 21005, Spain; Cardiology Department, Virgen del Rocío University Hospital, Manuel Siurot, s/n, Seville 41013, Spain; Cardiology Department, Virgen del Rocío University Hospital, Manuel Siurot, s/n, Seville 41013, Spain; Cardiology Department, Virgen del Rocío University Hospital, Manuel Siurot, s/n, Seville 41013, Spain; Cardiology Department, Virgen del Rocío University Hospital, Manuel Siurot, s/n, Seville 41013, Spain

**Keywords:** TAVI, Valve-in-valve, Bioprosthetic valve fracture, Ventricular septal defect, Percutaneous closure, Case report

## Abstract

**Background:**

Ventricular septal defect (VSD) is an unusual complication of transcatheter aortic valve implantation (TAVI). The risk factors are not well understood but may include oversizing, calcification amount and location, left-ventricular chamber morphology, and valve-in-valve (ViV) procedures. Percutaneous treatment is challenging but is usually the preferred option.

**Case summary:**

An 80-year-old woman with two previous surgical aortic valve replacements was admitted to our Cardiology Department for decompensated heart failure. New bioprosthesis degeneration (19 mm Mitroflow™, Sorin Group, Canada) was observed with severe intraprosthetic aortic regurgitation. After evaluation, the heart team chose to perform ViV TAVI. Because of the high risk of coronary obstruction, chimney stenting of both coronary arteries was performed. A 23 mm self-expandable Navitor™ valve (Abbott, IL, USA) was implanted, but the Mitroflow™ valve had to be cracked to minimize the persistent high gradient. During valve fracture, the non-compliant balloon broke and a small iatrogenic VSD appeared. However, the patient remained stable, so conservative management was selected. During follow-up, she developed severe haemolytic anaemia and heart failure; therefore, percutaneous closure of the iatrogenic VSD was performed twice, which was a difficult challenge.

**Discussion:**

A viable alternative to redo surgery is ViV TAVI. Risks include higher rates of prosthesis–patient mismatch and coronary obstruction. Occasionally, bioprosthetic valve fracture is required, particularly in small bioprostheses, to achieve low gradients. Anecdotally, fracture has led to annular rupture and VSD. Most VSDs are small and without clinical or haemodynamic repercussions; however, in symptomatic cases, percutaneous closure is a viable alternative to surgery.

**Learning points**

- Valve-in-valve transcatheter aortic valve implantation (TAVI) is a viable alternative for failed bioprosthetic surgical valves. Amongst the challenging problems related to this technique are coronary obstruction, especially in high-implant surgical prostheses, and residual transvalvular gradients, especially in small-size prostheses.
- Bioprosthetic valve fracture can be used to decrease patient–prosthesis mismatch, but complications, such as annular rupture and ventricular septal defect (VSD), may occur rarely.
- Iatrogenic VSD after TAVI is an unusual complication. Most VSDs are small with no clinical or haemodynamic repercussions, but they should be considered in patients with post-TAVR dyspnoea, right heart failure, or significant haemolysis.
- Percutaneous treatment of the VSD is preferred over open cardiac surgery because it avoids the high surgical risk in this patient population.

## Introduction

Transcatheter aortic valve implantation (TAVI) has a well-established role in treating symptomatic severe aortic stenosis, including high-, moderate-, and low-risk patients.^1^ There are many advantages of this technique, and the related complications include high-degree atrioventricular block, bleeding or thrombosis from vascular accesses, coronary obstruction, valve migration/embolization, and aortic root rupture.^2^ Iatrogenic ventricular septal defect (VSD) after TAVI is an unusual complication. Risk factors for this phenomenon are not well understood and may include oversizing, presence (location and amount) of calcification, morphology of the left-ventricular cavity, and the nature of the valve-in-valve procedures.^3^

Most VSDs are small, restrictive, have a small left-to-right shunt, and are without clinical or haemodynamic repercussions. Therefore, the diagnosis is usually made by echocardiography as an incidental finding, and no further interventions are required. However, this complication must be considered in patients with post-TAVR dyspnoea, right heart failure, or significant haemolysis. In symptomatic cases, percutaneous treatment is the preferred option in most of these patients because it avoids the high surgical risk.^3^

## Summary figure

**Table:** 

2002	Surgical aortic valve replacement (SAVR) using bioprostheses to treat severe degenerative aortic valve stenosis.
2012	Second SAVR using a 19 mm Mitroflow™ bioprosthesis to treat bioprosthetic degeneration (severe stenosis).
2021 (September)	Patient admitted to the hospital due to decompensated heart failure New prosthesis degeneration (severe regurgitation). Heart team evaluation. High surgical risk (Society of Thoracic Surgeons score of 7.8% and Euroscore II score of 8.2%). Valve-in-valve TAVI was selected. A 23 mm self-expandable Navitor™ valve was implanted with chimney stenting (high risk of coronary obstruction). Ring prosthesis fracture was performed (high gradient) with a high-pressure non-compliant balloon. The gradient disappeared but the balloon broke and a small and restrictive iatrogenic ventricular septal defect (VSD) was created. The VSD was well tolerated by the patient; therefore, initial conservative management was selected. She was discharged from the hospital 4 days after TAVI.
2021 (October)	New admission to hospital: severe haemolytic anaemia and heart failure Percutaneous closure of the iatrogenic VSD (6 mm Amplatzer™ muscular VSD occluder). Acceptable result with a residual shunt after the procedure. Due to the high risk of aortic, mitral, and tricuspid valves interactions, as well as valve damage, we decided to end the procedure and await for endothelialization of the device.
2021 (November)	The patient was readmitted to the hospital with the same symptoms of severe haemolytic anaemia and heart failure. A new percutaneous closure procedure was performed (12 × 9 mm Amplatzer™ Vascular Plug II). A negligible shunt persisted but was greatly reduced relative to the previous one. The patient was discharged from the hospital 4 days uneventfully.
2023 (June)	The patient has not required new hospital admissions or blood transfusions during the follow-up period and is New York Heart Association II functional class.

## Case presentation

An 80-year-old woman with two previous surgical aortic valve replacements (SAVRs) was admitted with decompensated heart failure. In 2002, she required SAVR because of severe degenerative aortic valve stenosis, and a bioprosthesis was implanted (the type and size of the prosthesis were unknown because the surgery was performed in another hospital). In 2012, due to degeneration of the bioprosthesis (severe stenosis), the patient required a new SAVR. In this case, a 19 mm Mitroflow™ biological valve (Sorin Group, Canada) was implanted, which failed in 2021 because of severe intraprosthetic regurgitation (mildly dilated left ventricle with normal ejection fraction).

The case was evaluated by the heart team, and valve-in-valve (ViV) TAVI was selected to avoid the high surgical risk (Society of Thoracic Surgeons score of 7.8%; Euroscore II score of 8.2%). Computed tomography (CT) before TAVI showed a high risk of coronary obstruction [high implantation of previous prosthesis and short distance to the origins of both coronary arteries: 3 mm for the left main (LM) and 5.8 mm for the right coronary artery (RCA), and a small virtual transcatheter heart valve to coronary distance (<4 mm)].

**Figure 1:**
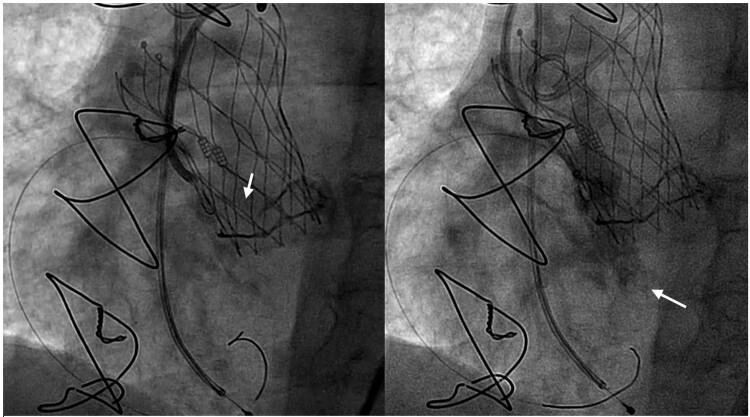
Bioprosthesis valve fracture. The left image shows a fractured prosthesis ring (arrow). The right image shows the shunt between left side and right sides (arrows).

A 23 mm self-expandable Navitor™ valve (Abbott, IL, USA) was implanted following the usual technique with chimney stenting of both coronary arteries (using a 4 × 23 mm and a 3 × 23 mm drug-eluting stents for the LM and the RCA, respectively). Valve post-dilation with a non-compliant (NC) balloon (and kissing balloon with both stents) was necessary because of the high residual gradient (30 mmHg), but it was insufficient. Therefore, a high-pressure NC balloon was used to fracture the Mitroflow™ prosthesis ring, which led to the disappearance of the gradient, but the balloon ruptured unfortunately, and a small shunt between the left and right side was observed (*Figure 1*; Supplementary material online, *Video S1*).

**Figure 2:**
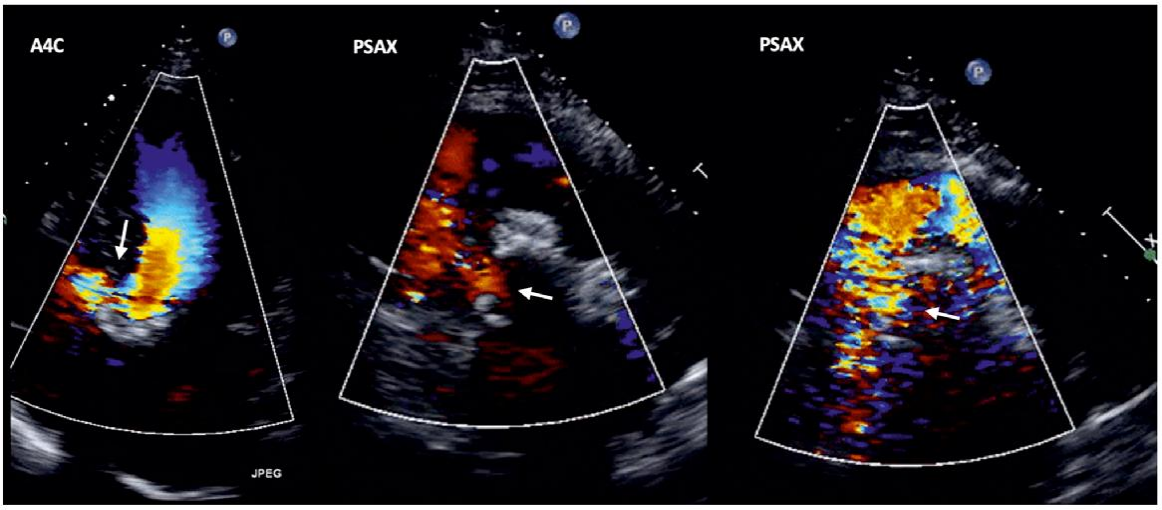
Iatrogenic ventricular septal defect detected by transthoracic echocardiography (Doppler colour). A4C, apical four-chamber; PSAX, parasternal short-axis base.

An iatrogenic VSD was suspected and confirmed by transthoracic echocardiography (TTE) (*Figure 2*). The VSD was small, restrictive (6 × 4 mm septal defect, 0.23 cm^2^ area, estimated peak velocity of 4.95 m/s, peak gradient of 94 mmHg, Qp/Qs of 1.4:1, and mildly elevated pulmonary artery systolic pressure of 39 mmHg by TTE), and well tolerated by the patient (the clinical exam revealed a new holosystolic murmur with a preserved second sound, without heart failure); therefore, initial conservative management was deemed appropriate. During her hospital admission, the patient required a permanent pacemaker because of a third-degree atrioventricular block. She was discharged from the hospital 4 days after TAVI.

**Figure 3:**
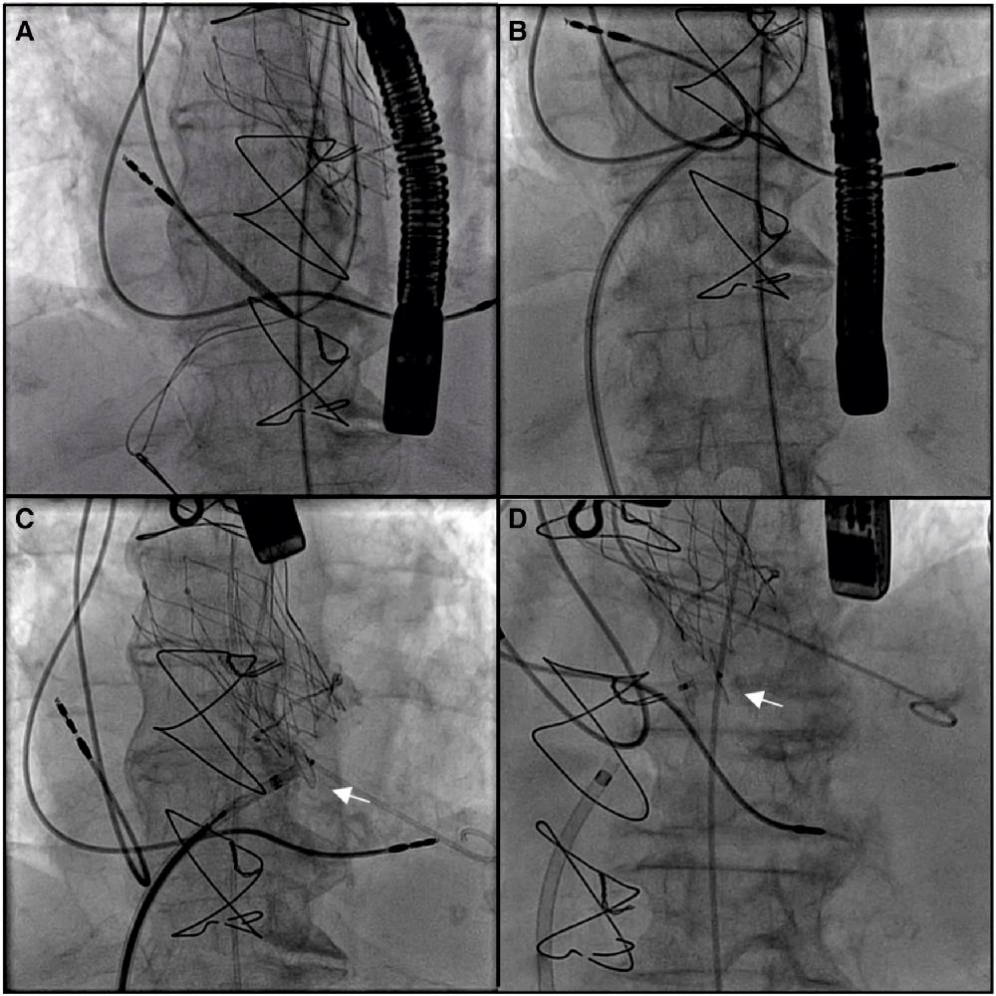
Closure of the iatrogenic ventricular septal defect with an Amplatzer™ muscular ventricular septal defect occluder. (*A*) Wire snaring after crossing the ventricular septal defect. (*B*) Amplatzer™ sheath advancing over the wire. (*C*) Shown is the 6 mm Amplatzer™ muscular ventricular septal defect occluder implantation (arrow). (*D*) The implanted Amplatzer™ muscular ventricular septal defect occluder (arrow).

During the follow-up, the patient developed severe haemolytic anaemia requiring blood transfusions. Heart failure occurred again (dyspnoea at rest, bibasal crackles, and elevated N-terminal pro B-type natriuretic peptide (NT-proBNP) 19 790 pg/mL; to rule out heart failure with a negative predictive value of 94–98%, the NT-proBNP value should be <300 pg/mL for acute heart failure and <125 pg/mL for chronic heart failure) for which she was readmitted to the hospital, and percutaneous closure of the iatrogenic VSD was scheduled (see Supplementary material online, *Video S2*). A hydrophilic wire and a Judkins right catheter were used to retrogradely cross the defect (from the left to the right side; previous failed antegrade crossing from the right side). The wire was snared with a gooseneck snare, exteriorized from the femoral vein, and an arteriovenous circuit was created. The Amplatzer™ (Abbott, IL, USA) sheath was advanced over the wire from the femoral vein to the ascending aorta. The selected device for VSD closure (6 mm Amplatzer™ muscular VSD occluder) was advanced to the tip of the sheath, and the entire system was withdrawn to the left ventricular outflow tract. The left disk was opened against the membranous septum, a gentle traction of the device was then performed, and the right disk was finally deployed, without any interaction with the leaflets (*Figure 3*; Supplementary material online, *Video S3*).

An acceptable result was achieved without immediate complications, but a residual shunt persisted after the procedure. Due to the high risk of aortic, mitral, and tricuspid valve interactions, it was decided to end the procedure and wait for proper endothelialization. However, after 2 weeks, the patient was readmitted to the hospital with the same symptoms (haemolytic anaemia and heart failure), so a second attempt was warranted.

**Figure 4:**
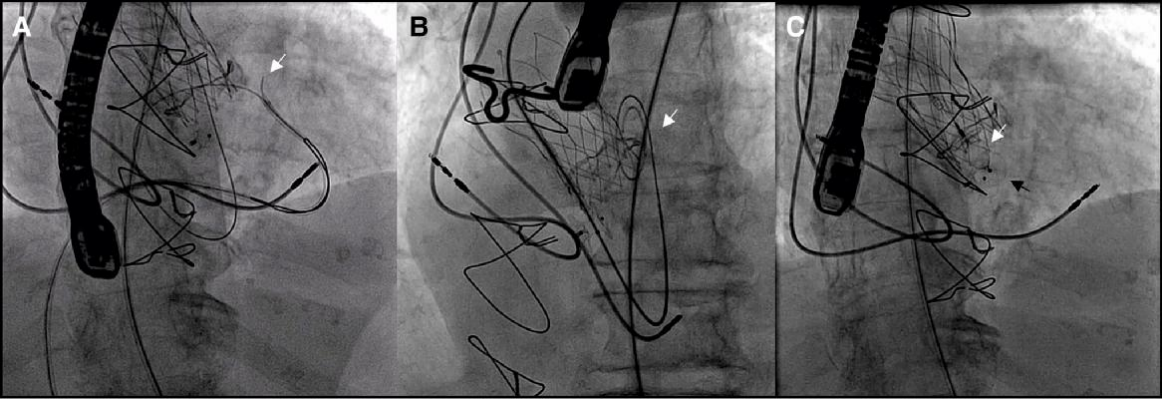
Closure of the iatrogenic ventricular septal defect (second procedure) with an Amplatzer™ Vascular Plug II. (*A*) Wire snaring after crossing the ventricular septal defect. (*B*) High support wire for device implantation. (*C*) Second device implantation (up arrow: Amplatzer™ Vascular Plug II; down arrow: Amplatzer™ muscular ventricular septal defect occluder).

**Figure 5:**
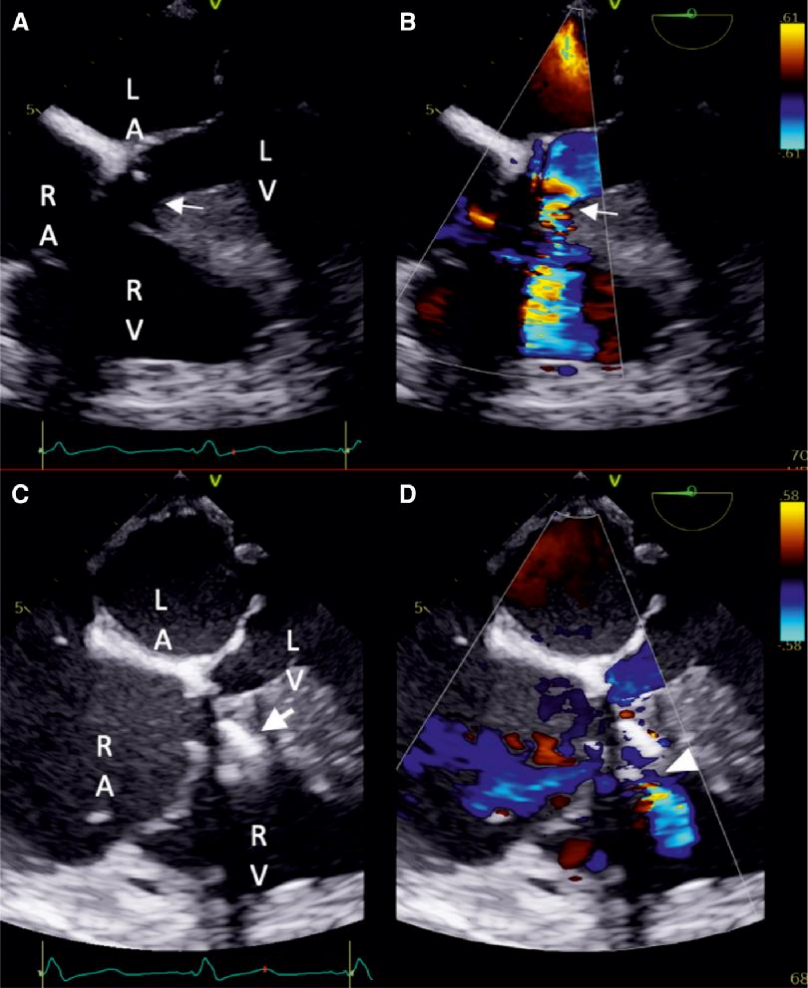
(*A*, *B*) Transoesophageal four-chamber view, 2D colour comparison showing the ventricular septal defect. Arrow: ventricular septal defect. (*C*, *D*) Transoesophageal four-chamber view, 2D colour comparison after ventricular septal defect closure. Arrow: ventricular septal defect closure device; arrowhead: residual ventricular septal defect shunt. LA, left atrium; LV, left ventricle; RA, right atrium; RV, right ventricle.

In the second procedure, the defect was retrogradely crossed again and a smaller device (12 × 9 mm Amplatzer™ Vascular Plug II) was used, without complications (*Figure 4*; Supplementary material online, *Video S4*). A negligible shunt still persisted, but we decided to end the procedure. Subsequently, the patient’s recovery was uneventful, and she was discharged after 4 days (*Figure 5*; Supplementary material online, *Videos S5 and S6*). She did not require new hospital admissions or blood transfusions during follow-up. The last cardiology visit was in May 2023 and the patient was asymptomatic.

## Discussion

Bioprostheses now represent the majority of SAVRs and are increasingly implanted in younger patients who are expected to the need future valve reintervention.^2^ Thus, ViV TAVI is a viable alternative for failed bioprosthetic surgical valves, although the technique has many challenges, including an increased potential risk of coronary obstruction in certain SAVR types, high residual transvalvular gradients, and valve thrombosis.^2^

In some cases, bioprosthetic valve fracture is necessary, especially in small bioprostheses, to achieve low gradients and decrease prosthesis–patient mismatch. To fracture the surgical valve ring, high-pressure NC balloon inflation is performed. Complications, such as annular rupture, iatrogenic VSD, and damage to the mitral valve or transcatheter valve, occur rarely.^4^

Ventricular septal defect is an unusual complication after TAVI, and the mechanism is unclear.^3,5^ In our case, the most likely explanation is that the NC balloon burst caused the VSD. Moreover, an accurate evaluation by CT of the aortic valve annulus and the calcifications before TAVI could be necessary in order to minimize the risk of complications.^6^

In symptomatic cases of iatrogenic VSD, such as ours, percutaneous closure is a viable alternative to surgery. In the literature, there are several cases in which haemodynamically significant iatrogenic VSD after TAVI was successfully closed with an Amplatzer™ occluding device.^3,7,8^ However, there are some concerns associated with this technique, such as valve displacement in the VSD closure device placement process or the interaction between the device and valve function. This treatment can present a significant challenge; therefore, it is crucial to thoroughly plan the procedure to achieve the best possible result and avoid complications or structural damage.^6,9^

## Supplementary Material

ytae045_Supplementary_Data

## Data Availability

The data underlying this study are available in the article and in its online Supplementary material.

## Lead author biography

Dr Alba Abril Molina completed her medical school education at the University of Extremadura (Badajoz, Spain), and she pursued her residency at Virgen del Rocío University Hospital in the Department of Cardiology (Seville, Spain). Her primary area of expertise includes interventional cardiology. She currently works as an Interventional Cardiologist at the Juan Ramón Jiménez University Hospital (Huelva), Spain.
